# Combination CTLA-4 immunoglobulin treatment and ultrasound microbubble-mediated exposure improve renal function in a rat model of diabetic nephropathy

**DOI:** 10.18632/aging.202664

**Published:** 2021-03-10

**Authors:** Liang Wang, Pengfei Wang, Xiuyun Li, Yanyan Dong, Senmin Wu, Maosheng Xu, Xiu Chen, Shijia Wang, Chao Zheng, Chunpeng Zou

**Affiliations:** 1 Department of Ultrasonic Diagnosis, The Second Affiliated Hospital and Yuying Children’s Hospital of Wenzhou Medical University, Wenzhou 325027, Zhejiang, China; 2 Department of Endocrinology, The Second Affiliated Hospital of Zhejiang University School of Medicine, Hangzhou 310000, Zhejiang, China

**Keywords:** cytotoxic T lymphocyte associated antigen 4 immunoglobulin, diabetic nephropathy, ultrasound, microbubble, sonoporation

## Abstract

Objective: This study explored the therapeutic impact of combined cytotoxic T lymphocyte-associated antigen 4 immunoglobulin (CTLA-4-Ig) treatment and microbubble-mediated exposure in a rat model of diabetic nephropathy (DN).

Method: We treated rats using CTLA-4-Ig and/or microbubble exposure. At 8 weeks post-intervention, key parameters were evaluated including blood biochemistry, damage to renal tissue, renal parenchymal elasticity, ultrastructural changes in podocytes, and renal parenchymal expression of CD31, CD34, IL-6, Fn, Collagen I, Talin, Paxillin, α3β1, podocin, nephrin, and B7-1.

Result: We found that renal function in the rat model of DN can be significantly improved by CTLA-4-Ig and CTLA-4-Ig + ultrasound microbubble treatment. Treatment efficacy was associated with reductions in renal parenchymal hardness, decreases in podocyte reduction, decreased IL-6, Fn and Collagen I expression, increased Talin, Paxillin and α3β1 expression, elevated podocin and nephrin expression, and decreased B7-1 expression. In contrast, these treatments did not impact CD31 or CD34 expression within the renal parenchyma.

Conclusion: These findings clearly emphasize that CTLA-4-Ig can effectively prevent podocyte damage, inhibiting inflammation and fibrosis, and thereby treating and preventing DN. In addition, ultrasound microbubble exposure can improve the ability of CTLA-4-Ig to pass through the glomerular basement membrane in order to access podocytes such that combination CTLA-4-Ig + microbubble exposure treatment is superior to treatment with CTLA-4-Ig only.

## INTRODUCTION

In many patients with diabetes, serious chronic complications such as diabetic nephropathy (DN) can develop [1, 2]. DN is characterized by diffuse glomerular sclerosis and proteinuria [3, 4], with the latter being directly linked to diabetes-associated podocyte injury and apoptotic death [5]. Antigen-presenting cells commonly express the immune protein B7-1 [6], and the upregulation of this protein in podocytes has been shown to be closely linked to proteinuria [7]. Cytotoxic T lymphocyte-associated antigen 4 immunoglobulin (CTLA-4-Ig) can suppress B7-1 activity, thereby preventing the development of autoimmune pathology [8, 9]. Treatment of DN model rats with CTLA-4-Ig has been shown to facilitate podocyte repair, to improve podocyte activity, and to alleviate proteinuria [10]. The glomerular filtration membrane, however, is made up of layers of endothelial cells and podocytes separated by the glomerular basement membrane [11, 12]. Circulating CTLA-4-Ig must therefore be capable of passing through both the endothelium and this basement membrane in order to access podocytes and to bind the B7-1 molecules expressed on these cells. To date, however, few studies have identified strategies for improving the ability of CTLA-4-Ig to pass through these barriers in order to bind to podocyte B7-1.

Ultrasonic microbubbles are a form of drug delivery system that represents an attractive alternative to adenoviral vectors or plasmids [13], allowing for targeted drug administration via a sonoporation process [14–16]. Microbubbles enable researchers to monitor the efficacy of such sonoporation in real-time at the site of treatment [17]. Importantly, prior work suggests that such ultrasound-mediated microbubbles can enhance renal interstitial capillary permeability in DN model rats [18].

The present study was designed to assess whether microbubble-induced sonoporation is capable of enhancing the ability of CTLA-4-Ig to penetrate the glomerular filtration membrane and to access podocytes, thereby improving the competitive inhibition of B7-1 and preventing podocyte detachment from the basement membrane. Specifically, we compared the relative efficacy of targeted microbubble-mediated CTLA-4-Ig delivery to that of treatment with CTLA-4-Ig alone in a rat model of DN. If successful, this approach may represent a viable strategy for the clinical treatment of DN patients throughout the world.

## RESULTS

### Assessment of key physiological and biochemical indicators in treated rats

**Figure 1:**
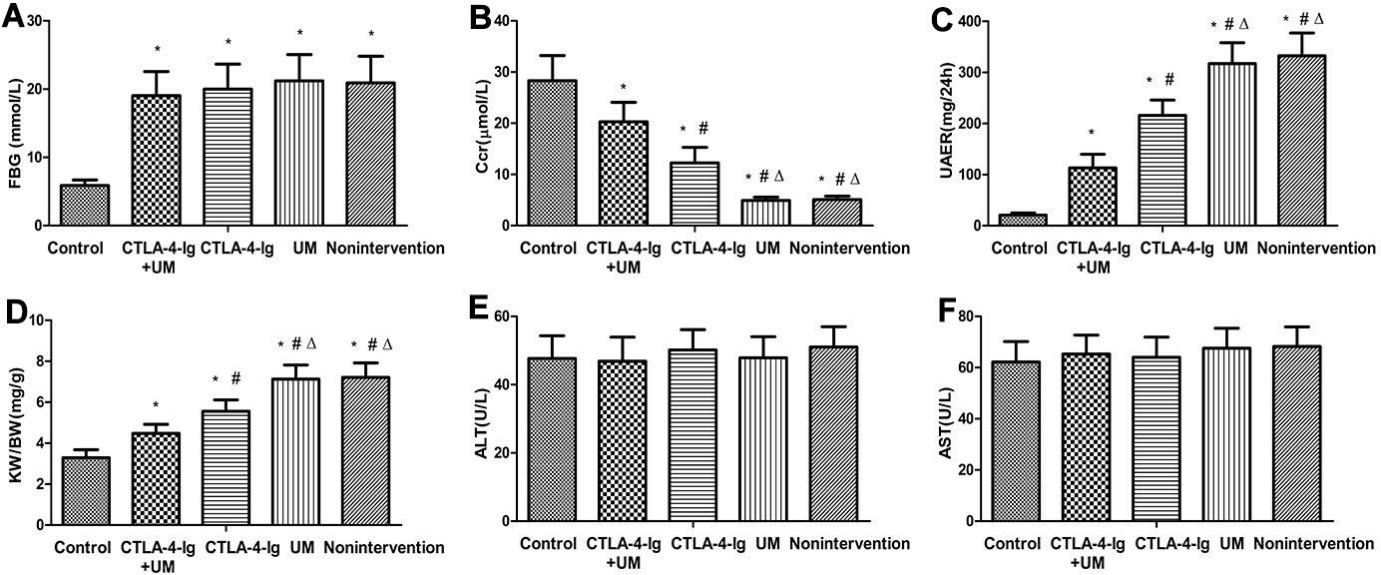
**Comparison of FBG, Ccr, UAER, KW / BW, ALT, and AST in five groups of rats.** Control: control group; CTLA-4-Ig+UM: CTLA-4-Ig+ ultrasound microbubble exposure group; CTLA-4-Ig: CTLA-4-Ig group; UM: ultrasound microbubble exposure group; Nonintervention: no intervention group. Data are means ± SD. (**A**) FBG: fasting blood glucose, * P <0.05 vs control; (**B**) Ccr: endogenous creatinine excretion rate, * P <0.05 vs control, #P <0.05 vs CTLA-4-Ig + UM, Δ P <0.05 vs CTLA -4-Ig; (**C**) UAER: 24-hour urine albumin excretion rate, * P <0.05 vs control, #P <0.05 vs CTLA-4-Ig + UM, Δ P <0.05 vs CTLA-4-Ig; (**D**) KW / BW: Glomerular hypertrophy index (kidney weight / body weight), * P <0.05 vs control, #P <0.05 vs CTLA-4-Ig + UM, Δ P <0.05 vs CTLA-4-Ig; (**E**) ALT: Alanine aminotransferase, no significant difference between groups; (**F**) AST: Aspartate aminotransferase, no significant difference between groups.

When we compared fasting blood glucose (FBG) levels among treatment groups, we found that these levels were comparable among the untreated model, CTLA-4-Ig-treated, microbubble-treated, and CTLA-4-Ig + microbubble-treated groups (groups B, C, D, and E, respectively; P>0.05), whereas levels in all four of these groups were higher than in the healthy control group (group A; P<0.05). Endogenous creatinine clearance (Ccr) values did not differ significantly between groups B and D (P>0.05), but the values in these two groups were significantly below those measured in the three other groups (P<0.05). Specifically, these Ccr values were the highest in group A, with progressively lower values in groups E and C. The 24h urinary albumin excretion rate (UAER) and glomerular hypertrophy index (kidney weight/ body weight, KW/BW) values similarly did not differ between groups B and D (P>0.05), whereas the values in these groups were markedly elevated relative to the three other treatment groups (P<0.05). These values were significantly higher in group C animals relative to animals in group E, with values in group A animals being significantly lower than those in group E. No differences in alanine aminotransferase (ALT) or aspartate aminotransferase (AST) levels were measured among these five groups (P>0.05) (Table 1 and Figure 1).

**Table 1:** Comparison of general indicators and biochemical indicators of rats in each group (x±S).

**Indicators**	**Control**	**CTLA-4-Ig+UM**	**CTLA-4-Ig**	**UM**	**Nonintervention**
FBG (mmol/L)	5.88±0.81	19.05±3.52^a^	20.02±3.65^a^	21.22±3.84^a^	20.92±3.90^a^
Ccr(ml/min)	28.33±4.89	24.02±5.12^a^	20.28±3.81^ab^	5.26±0.82^abc^	5.08±0.67^abc^
UAER(mg/24h)	20.69±4.13	113.32±26.42^a^	215.74±30.28^ab^	317.31±40.74^abc^	332.42±44.81^abc^
KW/BW(mg/g)	3.29±0.39	4.48±0.44^a^	5.56±0.55^ab^	7.13±0.69^abc^	7.21±0.71^abc^
ALT(U/L)	47.17±6.63	46.88±7.02	50.15±5.99	47.89±6.14	51.07±5.77
AST(U/L)	62.18±8.00	65.33±7.39	64.01±7.85	67.58±7.77	68.20±7.53

^a^*P*<0.05 compared with the control group; ^b^*P* <0.05 compared with the CTLA-4-Ig+ ultrasound microbubble exposure group; ^c^*P*<0.05 compared with the CTLA-4-Ig group. FBG, fasting blood glucose; Ccr, creatinine clearance rate; UAER, urinary albumin excretion rate; KW/BW, kidney weight/body weight; ALT, alanine aminotransferase; AST, aspartate aminotransferase.

### Assessment of renal elasticity scores

**Figure 2:**
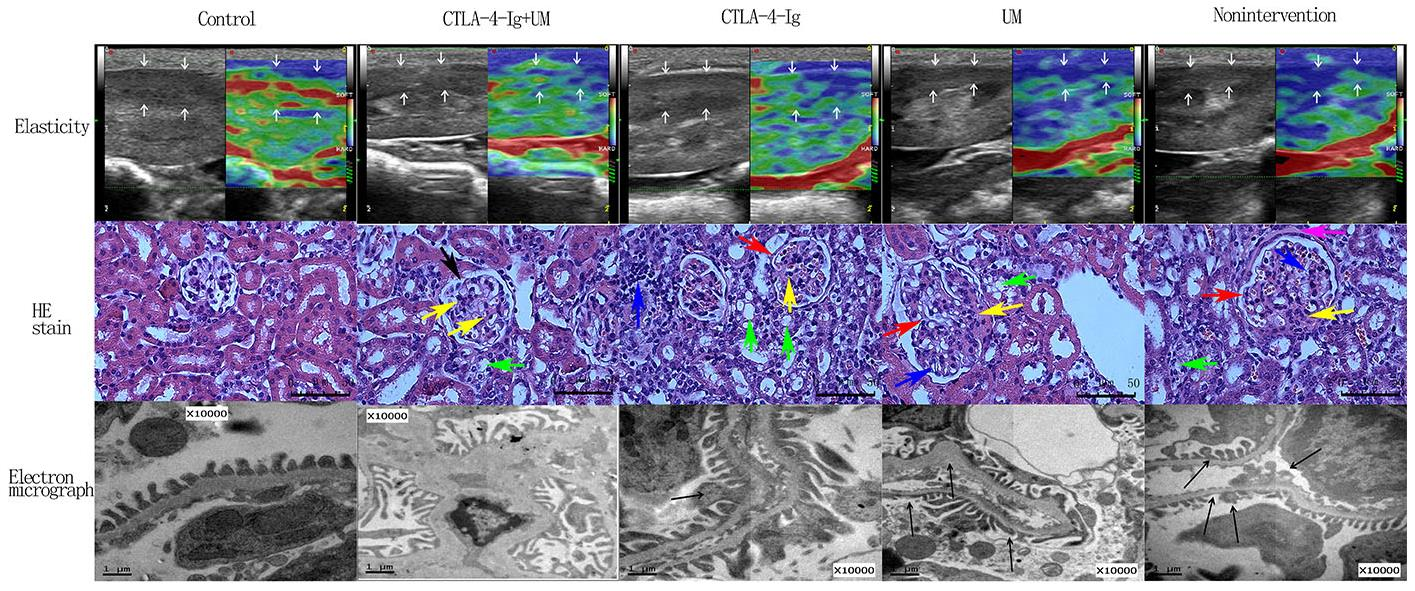
**Elastic imaging, H&E staining, and electron micrograph analyses of right kidney parenchymal podocytes in rats.** Assessment of rat renal parenchymal elasticity. Control group: The majority of the region of interest (arrow) is green, with a small portion being red; score = 1. CTLA-4-Ig + UM group: The majority of the region of interest (arrow) is green, with some areas being red and blue; score = 2. CTLA-4-Ig group: The majority of the region of interest (arrow) is green, with some areas being red and blue; score = 2. UM group: The region of interest (arrow) is primarily blue with some green; score = 3. Non-intervention group: The region of interest (arrow) is primarily blue with some green; score = 3. H&E staining of renal parenchymal tissue samples. -Control group: No glomerular capillary cavity changes, cellular proliferation, or basement membrane thickening are evident, with clear glomerular balloon; -CTLA-4-Ig + UM group: Glomerular volume is slightly enlarged and the glomerular basement membrane is partially thickened (black arrow), with slight cellular proliferation, with a small amount of hyaline substance deposition (yellow arrow) and vacuolated degeneration of the renal tubular epithelial cells (green arrow); CTLA-4-Ig group: Glomerular basement membrane thickening is evident (red arrow), with cellular proliferation, a small amount of hyaline substance deposition (yellow arrow), narrowing of the partial capillary lumen, marked vacuolated degeneration of the renal tubular epithelial cells (green arrow), and interstitial lymphocyte infiltration (blue arrow); UM group: Glomerular volume enlargement and basement membrane thickening are evident (red arrow), with marked cellular proliferation, flaky hyaline substance deposition (yellow arrow), vacuolated degeneration of renal tubular epithelial cells (green arrow), and narrowing of the capillary lumen (blue arrow); Non-intervention group: Glomerular volume enlargement and basement membrane thickening are evident (red arrow), with cellular proliferation, flaky hyaline substance deposition (yellow arrow), vacuolated degeneration of the renal tubular epithelial cells (green arrow), narrowing of the capillary lumen (blue arrow), and hyalinosis of the glomerular wall (pink arrow). Assessment of podocyte ultrastructural features via TEM. (The splicing is used to join together two parts of the same TEM image due to the limitation of the field of view). Control group: Podocytes exhibit a uniform arrangement without any fusion or loss; CTLA-4-Ig + UM group: Podocyte synapses appear disorderly, without obvious fusion or loss; CTLA-4-Ig group: Podocyte synapse structures are still present, but with visible evidence of fusion (black arrow); UM group: Disorder of the podocyte synapse is evident, with some missing synapses, slight protrusion of the basement membrane, and visible synaptic fusion; Non-intervention group: the volume of the podocyte synapse is larger, with some missing and fused podocytes.

We next assessed elasticity scores for rats in the different treatment groups (Figure 2). When comparing groups B and D, no differences in these scores were detected, suggesting that microbubbles alone had no therapeutic efficacy (P>0.05). In addition, scores in groups C and E did not differ significantly (CTLA-4-Ig vs. CTLA-4-Ig+ microbubble; P>0.05). In order from low to high, elasticity scores in these giver groups were as follows: group A < (E and C) < D and B (P<0.05) (Tables 2, 3 and Figure 2).

**Table 2:** Renal elasticity score of rats in each group.

**Score**	**Control**	**CTLA-4-Ig+UM**	**CTLA-4-Ig**	**UM**	**Nonintervention**
1	14	6	7	-	-
2	1	8	7	8	9
3	-	1	1	7	6

Control: control group; CTLA-4-Ig+UM: CTLA-4-Ig+ultrasound microbubble exposure group; CTLA-4-Ig: CTLA-4-Ig group; UM: ultrasound microbubble exposure group; Nonintervention: no intervention group.

**Table 3:** Comparison of renal elasticity score of rats in each group.

	control *vs* CTLA-4-Ig+UM	control *vs* CTLA-4-Ig	control *vs* UM
*Z*	-3.049	-2.751	-4.854
*P*	0.011*	0.026*	0.00*
	CTLA-4-Ig+UM *vs* CTLA-4-Ig	control *vs* nonintervention	CTLA-4-Ig+UM *vs* UM
*Z*	-0.326	-4.847	-3.156
*P*	0.775	0.000*	0.003*
	CTLA-4-Ig+UM *vs* nonintervention	CTLA-4-Ig *vs* UM	UM *vs* nonintervention
*Z*	-2.994	-3.295	-0.362
*P*	0.007*	0.002*	0.775
	CTLA-4-Ig *vs* nonintervention		
*Z*	-3.153		
*P*	0.003*		

Control: control group; CTLA-4-Ig+UM: CTLA-4-Ig+ultrasound microbubble exposure group; CTLA-4-Ig: CTLA-4-Ig group; UM: ultrasound microbubble exposure group; Nonintervention: no intervention group. *P < 0.05.

### Assessment of renal morphology

When we imaged H&E stained kidney tissues from animals in the different treatment groups, morphological changes were most pronounced among samples from animals in groups B and D, consistent with our above results. In contrast, less severe morphological alterations were detected in animals in groups C and E, suggesting that treatment with CTLA-4-Ig with or without microbubble exposure were associated with reduced pathological kidney damage in this DN model system (Figure 2).

### Analysis of podocyte ultrastructural changes

In line with our H&E staining results we found that podocyte ultrastructural changes were most evident in samples from groups B and D, while these changes were slightly reduced in samples from groups C and E (Figure 2).

### Assessment of kidney CD31, CD34, IL-6, Fn, collagen I, talin, paxillin and α3β1 expression

**Figure 3:**
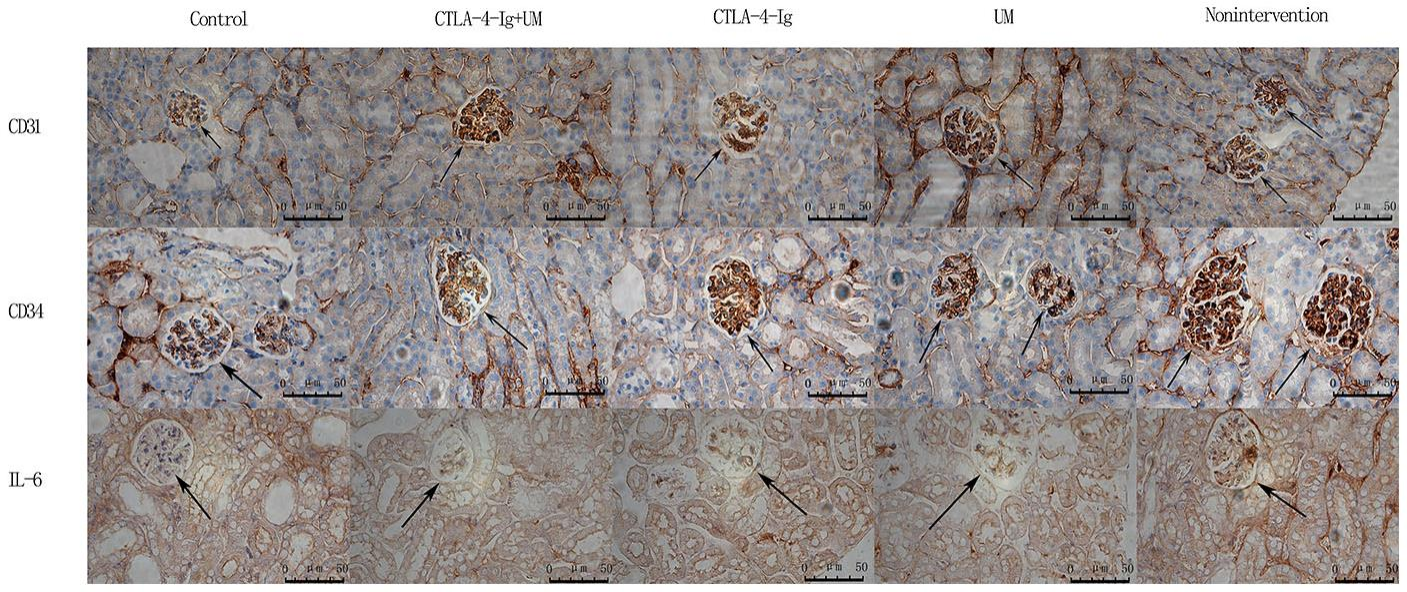
**Analysis of renal CD31, CD34, and IL-6 staining.** Analysis of renal CD31 staining. Control group: Weakly positive CD31 expression, with no evidence of blood vessel proliferation in the glomerulus (black arrow); CTLA-4-Ig + UM group: Strongly positive CD31 expression, with a small number of blood vessels in the glomerulus (black arrow); CTLA-4-Ig group: Strongly positive CD31 expression, with a small number of blood vessels in the glomerulus (black arrow); UM group: Strongly positive CD31 expression, with significant vascular proliferation in the glomerulus (black arrow); Non-intervention group: Strongly positive CD31 expression, with significant vascular proliferation in the glomerulus (black arrow). Analysis of renal CD34 staining. Control group: Weakly positive CD34 expression, with no evidence of blood vessel proliferation in the glomerulus (black arrow); CTLA-4-Ig + UM group: Strongly positive CD34 expression, with a small number of blood vessels in the glomerulus (black arrow); CTLA-4-Ig group: Strongly positive CD34 expression, with a small number of blood vessels in the glomerulus (black arrow); UM group: Strongly positive CD34 expression, with significant vascular proliferation in the glomerulus (black arrow); Non-intervention group: Strongly positive CD34 expression, with vascular hyperplasia in the glomerulus (black arrow). Analysis of renal IL-6 staining. Control group: Negative IL-6 expression in the glomerulus (black arrow); CTLA-4-Ig + UM group: Weakly positive IL-6 expression in the glomerulus (black arrow); CTLA-4-Ig group: Weakly positive IL-6 expression in the glomerulus (black arrow); UM group: Positive IL-6 expression in the glomerulus (black arrow); Non-intervention group: Strongly positive IL-6 expression in the glomerulus (black arrow).

**Figure 4:**
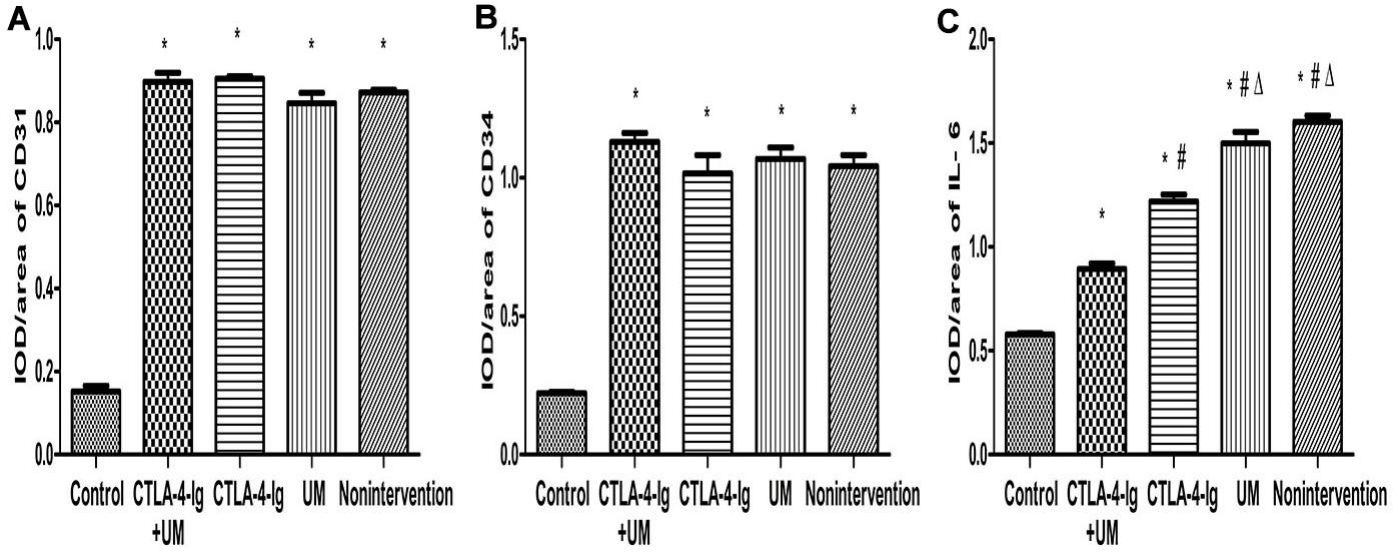
**Comparison of the expression of CD31, CD34, and IL-6 in rat kidneys.** Data are expressed as means ± standard deviation, IOD / area of CD31, CD34, and IL-6: integrated optical density value per unit area of CD31, CD34, and IL-6. (**A**) CD31, * P <0.05 vs control; (**B**) CD34, * P <0.05 vs control; (**C**) IL-6, * P <0.05 vs control, #P <0.05 vs CTLA-4-Ig + UM, Δ P <0.05 vs CTLA-4-Ig.

No significant differences in CD31 or CD34 expression were detected among the four DN treatment groups (groups B-E) (P>0.05), whereas the expression of both of these proteins was higher in all four groups relative to the healthy control group A (P<0.05) (Figures 3, 4).

**Figure 5:**
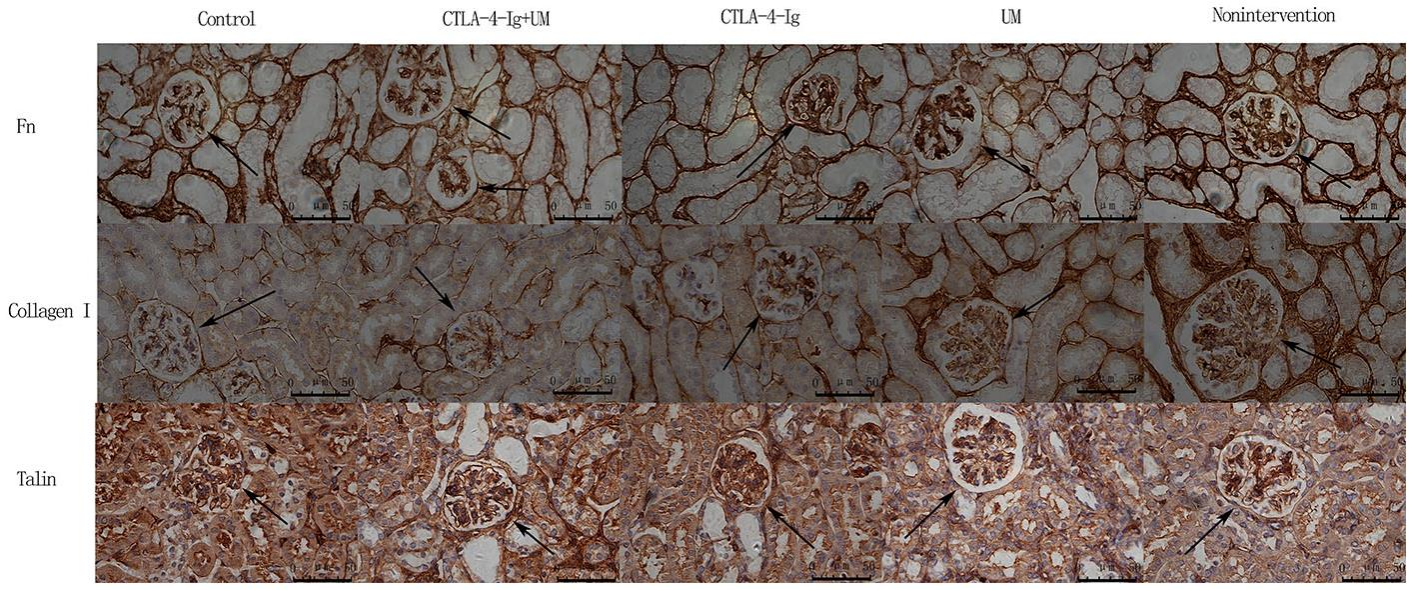
**Analysis of renal Fn, collagen I, and talin staining.** Analysis of renal Fn staining. Control group: Weakly positive Fn expression in the glomerulus (black arrow); CTLA-4-Ig + UM group: Positive Fn expression in the glomerulus (black arrow); CTLA-4-Ig group: Positive Fn expression in the glomerulus (black arrow); UM group: Strongly positive Fn expression in the glomerulus (black arrow); Non-intervention group: Strongly positive Fn expression in the glomerulus (black arrow). Analysis of renal Collagen I staining. Control group: Weakly positive Collagen I expression in the glomerulus (black arrow); CTLA-4-Ig + UM group: Weakly positive Collagen I expression in the glomerulus (black arrow); CTLA-4-Ig group: Weakly positive Collagen I expression in the glomerulus (black arrow); UM group: Positive Collagen I expression in the glomerulus (black arrow); Non-intervention group: Strongly positive Collagen I expression in the glomerulus (black arrow). Analysis of renal Talin staining. Control group: Strongly positive Talin expression in the glomerulus (black arrow); CTLA-4-Ig + UM group: Strongly positive Talin expression in the glomerulus (black arrow); CTLA-4-Ig group: Strongly positive Talin expression in the glomerulus (black arrow); UM group: Positive Talin expression in the glomerulus (black arrow); Non-intervention group: Positive Talin expression in the glomerulus (black arrow).

**Figure 6:**
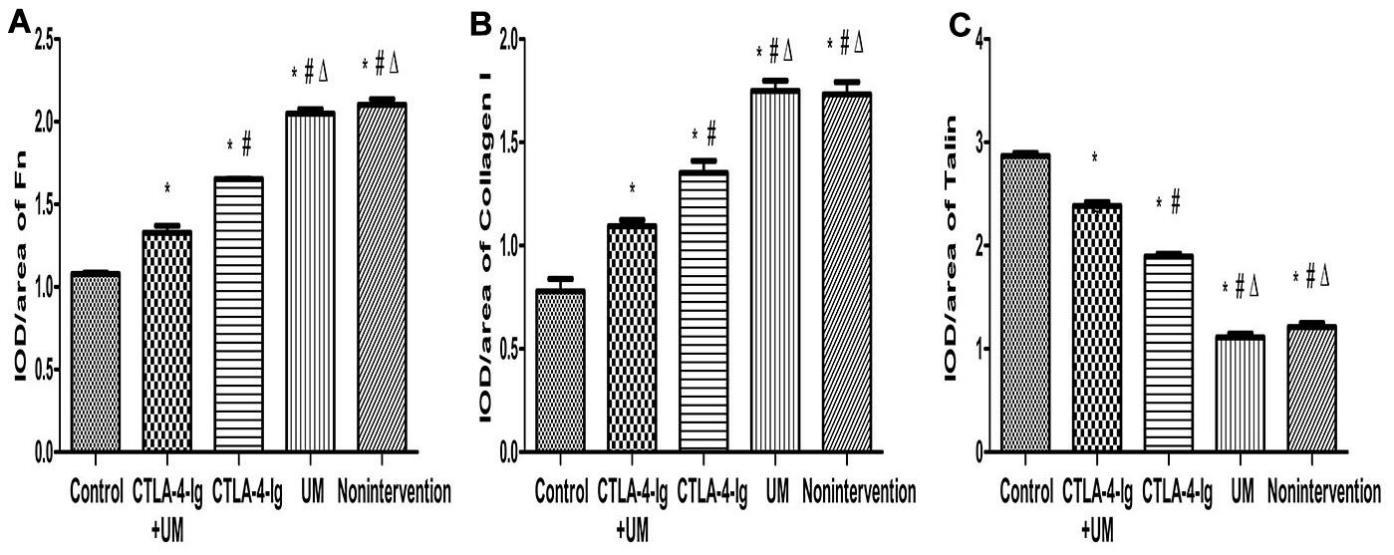
**Comparison of the expression of Fn, collagen I, and talin in rat kidneys.** Data are expressed as means ± standard deviation, IOD / area of Fn, Collagen I, and Talin: integrated optical density value per unit area of Fn, Collagen I, and Talin. (**A**) Fn, * P <0.05 vs control, #P <0.05 vs CTLA-4-Ig + UM, Δ P <0.05 vs CTLA-4-Ig; (**B**) Collagen I, * P <0.05 vs control, #P <0.05 vs CTLA-4-Ig + UM, Δ P <0.05 vs CTLA-4-Ig; (**C**) Talin, * P <0.05 vs control, #P <0.05 vs CTLA-4-Ig + UM, Δ P <0.05 vs CTLA-4-Ig.

We observed significant differences in IL-6 (Figures 3, 4), Fn, and Collagen I (Figures 5, 6) expression among these five treatment groups, with relative expression levels being, from low to high: group A < E < C < D and B (P<0.05). The differences between groups B and D were not significant (P<0.05).

**Figure 7:**
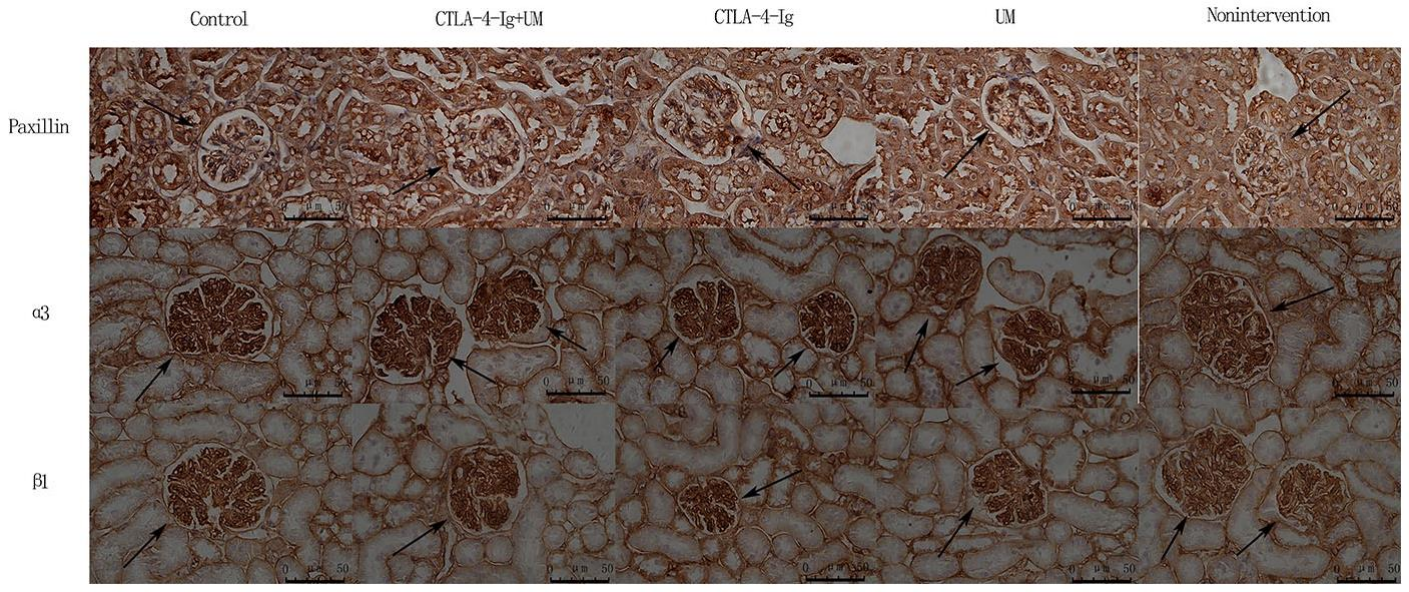
**Analysis of renal Paxillin, α3, and β1 staining.** Analysis of renal Paxillin staining. Control group: Strongly positive Paxillin expression in the glomerulus (black arrow); CTLA-4-Ig + UM group: Strongly positive Paxillin expression in the glomerulus (black arrow); CTLA-4-Ig group: Strongly positive Paxillin expression in the glomerulus (black arrow); UM group: Positive Paxillin expression in the glomerulus (black arrow); Non-intervention group: Positive Paxillin expression in the glomerulus (black arrow). Analysis of renal α3 staining. Control group: Strongly positive α3 expression in the glomerulus (black arrow); CTLA-4-Ig + UM group: Strongly positive α3 expression in the glomerulus (black arrow); CTLA-4-Ig group: Strongly positive α3 expression in the glomerulus (black arrow); UM group: Positive α3 expression in the glomerulus (black arrow); Non-intervention group: Positive α3 expression in the glomerulus (black arrow). Analysis of β1 staining. Control group: Strongly positive β1 expression in the glomerulus (black arrow); CTLA-4-Ig + UM group: Strongly positive β1 expression in the glomerulus (black arrow); CTLA-4-Ig group: Strongly positive β1 expression in the glomerulus (black arrow); UM group: Positive β1 expression in the glomerulus (black arrow); Non-intervention group: Positive β1 expression in the glomerulus (black arrow).

**Figure 8:**
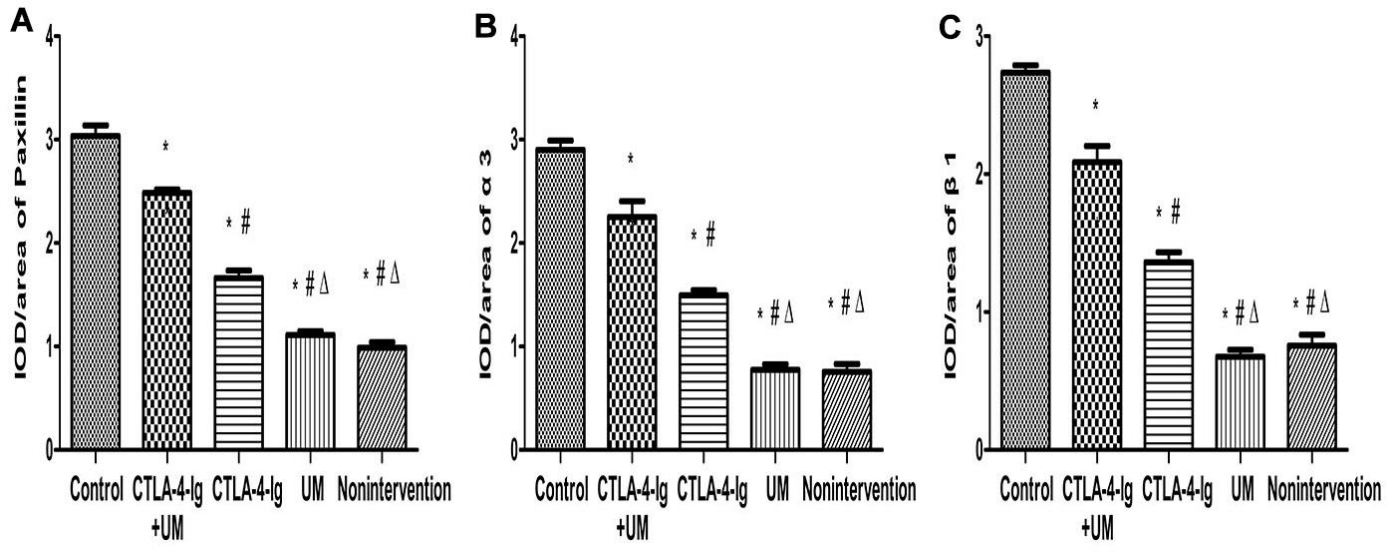
**Comparison of the expression of Paxillin, α3, and β1 in rat kidneys.** Data are expressed as means ± SD, IOD / area of Paxillin, α3 and β1: integrated optical density value per unit area of Paxillin, α3 and β1. (**A**) Paxillin, * P <0.05 vs control, #P <0.05 vs CTLA-4-Ig + UM, Δ P <0.05 vs CTLA-4-Ig; (**B**) α3, * P <0.05 vs control, #P <0.05 vs CTLA-4-Ig + UM, Δ P <0.05 vs CTLA-4-Ig; (**C**) β1, * P <0.05 vs control, #P <0.05 vs CTLA-4-Ig + UM, Δ P <0.05 vs CTLA-4-Ig.

We observed significant differences in Talin-1(Figures 5, 6), Paxillin and α3β1 (Figures 7, 8) expression among these five treatment groups, with relative expression levels being, from low to high: group D and B < C < E < A (P<0.05). The differences between groups B and D were not significant (P<0.05).

### Assessment of renal podocin, nephrin, and B7-1 protein levels

**Figure 9:**
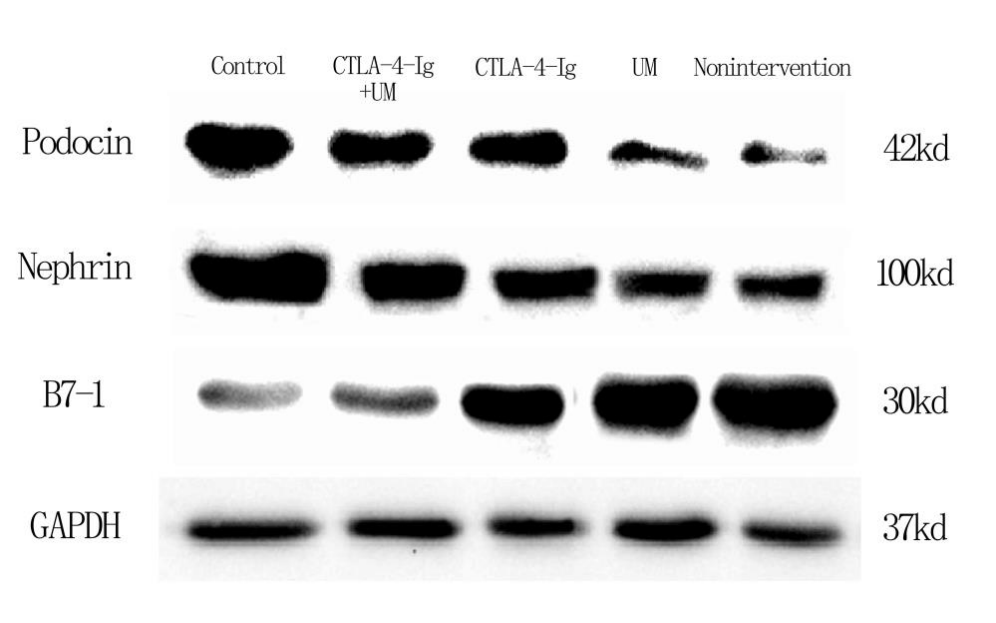
**Expression of podocin, nephrin, and B7-1 proteins in rat kidneys with west-blot.**

**Figure 10:**
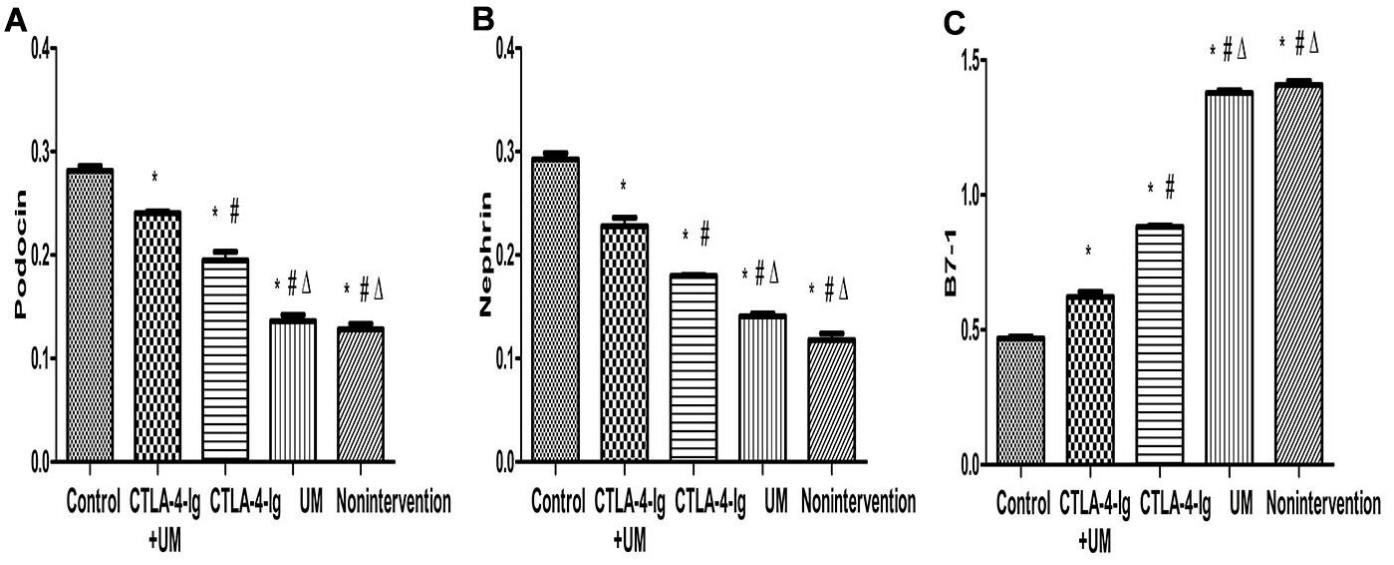
**Comparison of expression of podocin, nephrin, and B7-1 proteins in rat kidneys.** Data are expressed as means ± SD. IOD / area of podocin, nephrin, and B7-1: integrated optical density value per unit area of podocin, nephrin, and B7-1. (**A**) podocin, * P <0.05 vs control, #P <0.05 vs CTLA-4-Ig + UM, Δ P <0.05 vs CTLA-4-Ig; (**B**) nephrin, * P <0.05 vs control, #P <0.05 vs CTLA-4-Ig + UM, Δ P <0.05 vs CTLA-4-Ig; (**C**) B7-1, * P <0.05 vs control, #P <0.05 vs CTLA-4-Ig + UM, Δ P <0.05 vs CTLA-4-Ig.

We observed significant differences in podocin and nephrin expression among these five treatment groups, with relative expression levels being, from high to low: group A > E > C > D and B (P<0.05). The differences between groups B and D were not significant (P>0.05) (Figures 9, 10).

We additionally found that B7-1 protein levels varied among these five treatment groups, with these relative levels being, from low to high: group A < E < C < D and B (P<0.05). Differences between groups B and D were not significant (P>0.05) (Figures 9, 10).

## DISCUSSION

**Figure 11:**
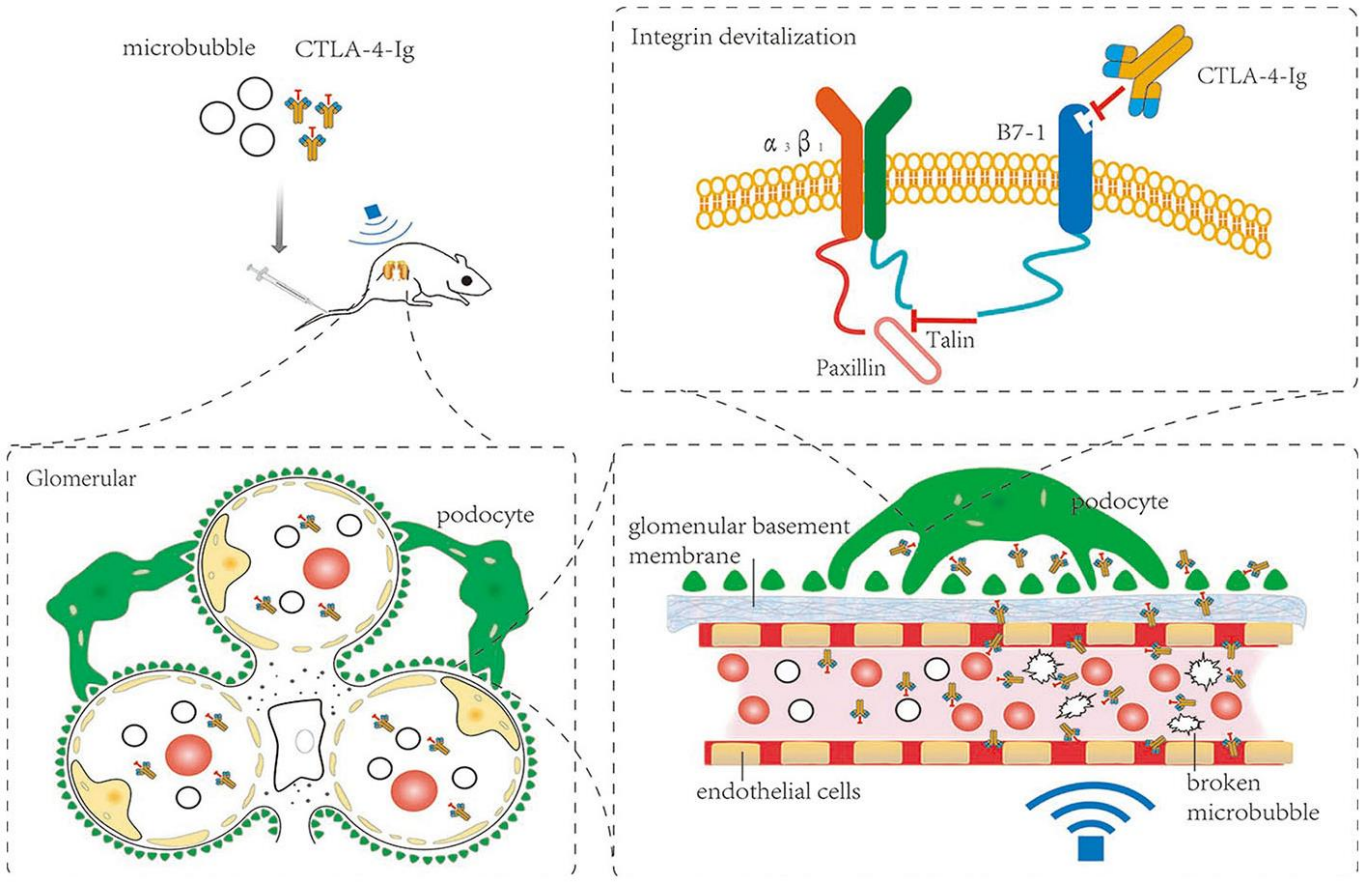
**Schematic overview of the present study.**

We utilized a rat model of DN and treated these animals with CTLA-4-Ig and/or microbubble exposure in order to explore the therapeutic efficacy of these approaches (Figure 11). None of these treatments significantly impacted blood glucose levels in these DN model animals. However, treatment with CTLA-4-Ig or CTLA-4-Ig + microbubble exposure significantly improved renal function, with the combination treatment being the most efficacious. Microbubble exposure alone had no effect on renal function. ALT and AST levels did not differ significantly among groups (P>0.05), indicating that these treatments did not impact liver function in DN rats. We also found that both CTLA-4-Ig and CTLA-4-Ig + microbubble exposure improved renal elasticity to a comparable degree, whereas microbubble exposure alone had no effect.

CD31 is known as platelet-endothelial cell adhesion molecule, and is a member of the immunoglobulin superfamily [19]. New blood vessels can form in glomeruli and interstitial cells in those with DN, and the growth of these vessels correlates with increased CD31 expression [20–22]. CD34 is distributed in the renal glomeruli and interstitium in rats [23]. Electron microscopy analyses of renal capillary vessels have established that CD34 molecules are concentrated on membrane processes, and the role of these structures in glomerular permeability warrants further study [24, 25]. Previous research has revealed that the expression of CD34 increases in the glomerular plasma membrane of diabetic animals [26]. Increased glomerular CD34 expression is related to age and diabetes [27]. In our study, CD31 and CD34 expression was significantly higher in all DN model mice relative to healthy control animals (P<0.05), but no differences in these expression levels were detected among treatment groups. This suggests that these treatments did not significantly improve glomerular endothelial cells in DN rats.

IL-6 is an important proinflammatory cytokine that can be secreted by B cells, keratinocytes, mononuclear macrophages and other cells. IL-6 plays a key role in the progression of DN through inflammatory response induction [28], and is a sensitive marker of DN development [29]. Sustained production of proinflammatory cytokines leads to increasingly severe local tissue inflammatory infiltration, which further degrades renal function. Fn is one of the most important components of the extracellular matrix (ECM), as increases in Fn levels will cause ECM agglomeration and accelerated DN development [30]. DN can be effectively treated by inhibiting Fn [31]. Collagen I is another key renal ECM component, levels of which rise in the context of DN such that the effective inhibition of collagen I can alleviate kidney damage [32, 33]. Herein, we found that IL-6, Fn, and Collagen I levels were decreased in the CTLA-4-Ig and CTLA-4-Ig + microbubble exposure treatment groups. This suggests that these CTLA-4-Ig-based treatments can inhibit inflammation and fibrosis, with combination therapy being superior to treatment with CTLA-4 alone, and with microbubble exposure not being protective.

Up to now, the mechanism of CTLA-4-Ig in the treatment of DN is unclear. There is a hypothesis: podocytes can be anchored to glomerular basement membrane(GBM) due to interactions between integrin α3β1 and talin which is a cytoskeleton-associated protein within podocytes under normal condition. But the interaction between talin and integrin α3β1 can be destroyed by B7-1 which was overexpressed in podocytes in DN [34, 35]. Because B7-1 can competitively bind to talin. As a result, the connection between podocytes and GBM become unstable, which lead to impaired glomerular filtration barrier and proteinuria. On the other hand, CTLA-4-Ig can inhibit the combination between B7-1 and to talin through competitively binding to B7-1, which facilitates the interaction between talin and integrin α3β1. Then podocytes became stable followed by repairing glomerular filtration barrier and relieving proteinuria [36]. In addition, studies have shown that paxillin may also participate in the above process and play an important role in the structure and function of podocytes [37, 38]. In our study, the expression of talin, paxillin and α3β1 was increased in these CTLA-4-Ig and CTLA-4-Ig + microbubble exposure treatment groups. This suggests that these CTLA-4-Ig-based treatments can incease expression of these three proteins, and furtherly enhance the stability of podocyte and decrease albuminuria, with combination therapy being superior to treatment with CTLA-4 alone, and with microbubble exposure not being protective.

Nephrin and podocin are podocyte-specific markers, the expression of which is significantly reduced in diabetic podocytes [39–41]. Proteinuria and DN are correlated with the reduced expression of nephrin and podocin [42]. Changes in expression of nephrin and podocin may alter the permeability of the glomerular filtration membrane. Nephrin expression is a potent predictor of DN, and the presence of nephrin in the urine strongly suggests that DN is developing in a given patient [43]. DN is a common driver of kidney failure in diabetes patients [44, 45], and is associated with B7-1 upregulation in podocytes [46]. Inhibition of B7-1 has been previously proposed to be an effective means of treating or preventing DN [47, 48]. Upregulation of B7-1 on podocytes coincides with the impairment of renal function, and CTLA-4-Ig can reverse such impairment as well as B7-1 expression [47, 48]. CTLA-4-Ig administration reduces rates of podocyte death, preventing further renal damage. These podocytes, however, are found on the outermost layer of the glomerular filtration membrane. Circulating CTLA-4-Ig therefore needs to pass through both glomerular capillary endothelial cells and the basement membrane in order to access B7-1 on podocytes. We therefore sought to optimize CTLA-4-Ig delivery to podocytes by increasing its ability to penetrate through endothelial cells and the basement membrane. In our study, the expression of podocin and nephrin was increased CTLA-4-Ig and CTLA-4-Ig + microbubble exposure treatments on podocytes, whereas B7-1 expression was decreased in these treatment groups. This suggests that these CTLA-4-Ig-based treatments can provide effective protection to podocytes, with combination therapy being superior to treatment with CTLA-4 alone, and with microbubble exposure not being protective.

Microbubbles are made up of external shells with gases interior [49, 50], enhancing microbubbles contrast in response to sound and allowing them to carry genes or drugs for targeted delivery [50, 51]. Ultrasonic energy can direct microbubble movement, and this combined microbubble and ultrasound treatment strategy can significantly improve local drug concentrations or gene expression [52–56]. Microbubbles are also valuable for imaging uses, and have been found to improve renal interstitial capillary permeability in DN model rats [18]. Indeed, the use of microbubbles with appropriate acoustic parameters has been shown to improve local vascular permeability without disrupting local vasculature [57, 58]. Such approaches to increasing cell membrane permeability are known as sonoporation, and rely upon acoustic cavitation for targeted drug or gene delivery and improved treatment efficacy [16, 59–62].

We treated DN model rats with CTLA-4-Ig and/or microbubble exposure. Following an 8-week treatment period, we found that combination CTLA-4-Ig + microbubble treatment significantly improved renal function, inhibited podocyte reduction, and improved the elasticity of the renal parenchyma. This combination therapy did not significantly alter renal CD31 or CD34 expression, though it did significantly decrease IL-6, Fn, and Collagen I expression, meanwhile increase Talin-1, Paxillin, α3β1, podocin and nephrin expression and it reduced B7-1 expression.

There are some limitations in this study. Firstly, we did not use different doses of CTLA-4-Ig to treat DN in our animal model experiments. Secondly, we did not assess the impact of different ultrasonic parameters on CTLA-4-Ig delivery. Thirdly, we did not conduct molecular mechanistic studies. We are potentially considering to pursue future experiments to study the molecular mechanism and provide useful information regarding how ultrasound can guide CTLA-4-Ig utilization for DN clinical treatment.

In conclusion, our results suggest that microbubble exposure and sonoporation may be able to enhance the therapeutic efficacy of CTLA-4-Ig via enhancing the passage of this antibody through the glomerular endothelium and basement membrane, allowing it to more readily access podocytes. Importantly, this combination CTLA-4-Ig + microbubble exposure treatment was superior to CTLA-4-Ig alone as a means of reducing DN-related renal pathology. These findings thus highlight a novel, safe, and efficacious approach to treating DN.

## MATERIALS AND METHODS

The Institutional Review Board of Wenzhou Medical University approved this study, and the Committee on Ethical Use of Animals at Wenzhou Medical University approved all animal work described herein.

### Reagents

A MyLab 60 device (Esaote, Genova, Italy) with a 4-13 MHz transducer (LA523) was used for color Doppler ultrasonography. We additionally utilized a light microscope (Nikon, Japan), a transmission electron microscope (TEM; H-600; Hitachi, Japan), CTLA-4-Ig (Abcam, UK), Streptozotocin (Sigma, USA), a urine protein quantitative detection kit (CBB method), a creatinine detection kit (picric acid method), a colorimetric alanine aminotransferase detection kit (Shanghai Jining Industrial Co., Ltd), a colorimetric aspartate transaminase detection kit (Shanghai Jianglai Biotech Co., Ltd), anti-CD31 (A0378, Abclonal), anti-CD34 (ab8158, Abcam), anti-IL6 (ab208113, Abcam), anti-Fibronectin (ab268021, Abcam), anti-Collagen I (A16891, Abclonal), anti-Talin 1 (ab71333, Abcam), anti-Paxillin (ab32084, Abcam), anti-Integrin alpha 3 (AF5182, Affinity Biosciences), anti-Integrin beta1 (ab179471, Abcam), anti-nephrin, anti-podocin, and anti-B7-1 (Shanghai Boyun Biotech Co., Ltd).

### Ultrasonic microbubble suspension preparation

SonoVue sulfur hexafluoride microbubbles were used for this study. Per bottle (59 mg of sulfur hexafluoride gas and 25 mg of a lyophilized powder), 5 mL sterile physiological saline (0.9% NaCl) was added. Bottles were then shaken thoroughly to yield a microbubble suspension in which the diameter of 90% of the microbubbles therein was < 6 μm (average = 2.5 μm). Prepared solutions were used within 6 hours, and were shaken immediately prior to use.

### CTLA-4-Ig preparation

Sterile physiological saline was used to resuspend lyophilized CTLA-4-Ig (1 mg per 2 mL), yielding a 0.5 mg/mL solution.

### CTLA-4-Ig + microbubble suspension preparation

Rather than being resuspended using physiological saline, lyophilized CTLA-4-Ig was resuspended in an ultrasonic microbubble suspension prepared as above at a 0.5 mg/mL concentration. Which ensured that CTLA-4-Ig-treated, microbubble-treated, and CTLA-4-Ig + microbubble-treated groups received the same amount of physiological saline but different amounts of CTLA-4-Ig and microbubbles. This solution was allowed to rest for 10-20 minutes at room temperature with repeated shaking prior to use.

### Animal treatment

For this study, male Sprague Dawley (SD) rats (6-8 weeks old; 200 ± 20 g) from Beijing Vital River Laboratory Animal Technology Co., Ltd. were used. In order to model DN, animals were fed a high-fat high-sugar diet (65% convention chow supplemented with 10 % cooked lard, 20% sucrose, 3% cholesterol, and 2% cholate), with 2% Streptozotocin (STZ) being administered to rats after four weeks. STZ was prepared using a citric acid buffer (pH = 4.2-4.5; 2.1 g citric acid in 100 mL ddH_2_O combined at a 1.32:1 ratio with a solution of 2.94 g sodium citrate in 100 mL ddH_2_O). STZ powder was dissolved using filtered 0.1 mol/L citric acid to prepare a 2% solution that was intraperitoneally injected into rats (30 mg/kg). At three days post-injection, a tail vein blood sample was collected from each animal after a 12 h fast to assess blood glucose levels. Blood glucose values ≥16.7 mmol/L were considered to be indicative of diabetes.

In total we utilized 75 rats that were randomized into 5 groups (n=15/group): Control rats that were fed regular food and water without treatment (Group A); an untreated model group in which DN was established based on prior approaches [63], with rats being maintained on a high-far high-sugar diet for 8 additional weeks without any treatment (Group B); a CTLA-4-Ig group in which DN was established as above, and rats were fed a high-fat high-sugar diet for 8 weeks during which time they were intravenously administered 0.5 mg/kg CTLA-4-Ig per week via tail vein injection (Group C); A microbubble exposure group in which DN was established as above, rats were maintained on a high-fat high-sugar diet for 8 weeks during which they SonoVue tail vein injections (1 ml/kg/week) with simultaneous ultrasound exposure to the kidneys (Group D); A combination CTLA-4-Ig + ultrasound microbubble exposition group wherein rats were treated as in Group D, but were injected with both CTLA-4-Ig (0.5 mg/kg/w) and SonoVue (1 ml/kg/w) while undergoing ultrasound exposure (Group E). The rats’ kidneys were exposed to 1 MHz ultrasound waves for 4 minutes per exposure twice per week with the following settings: 100 Hz pulse repetition frequency, 1.5 W/cm^2^ output intensity, 0.2 MPa peak negative acoustic pressure and 60% duty cycle [64]. Animals had free access to food and water at all times and were not treated with insulin or other hypoglycemic agents.

### Assessment of renal parenchymal elasticity

One day prior to euthanasia, all animals underwent an ultrasound-based assessment conducted by a single sonographer. Briefly, hair was removed from the lower back of each rat, the surface was cleaned, and an ultrasonic coupling agent was applied. An ultrasound probe was then positioned at an appropriate angle in contact with this region such that a maximal longitudinal section of the right kidney was visible in 2D grayscale mode. The elastic imaging mode was then activated, and a parenchymal elasticity score for the right kidney was measured based on the scoring system previously detailed by Itoh et al [65].

### Assessment of blood and urine biochemistry

At 24 hours prior to experimental termination, urine was collected from all animals. Total urine volumes were measured and used to calculate the urine volume per minute (UVPM). These samples were then spun for 10 minutes at 3500 rpm prior to being stored at -80° C for measurements of 24h UAER and urine creatinine (Ucr) levels. The body weight (BW) of each rat was measured prior to sacrifice. Animals were then anesthetized using chloral hydrate (400 mg/kg, i.p.). Blood was then collected from the right common carotid artery of each animal, and was spun for 10 minutes at 3500 rpm at 4° C before storage at -20° C for measurements of FBG, ALT, AST, and serum creatinine (Scr). In addition, Ccr was calculated as follows: Ccr = (Ucr × UVPM) / Scr, where UVPM = 24 h urine volume/ (24 × 60). Immediately following blood collection, kidney tissues were collected from each rat.

### Assessment of kidney morphology

### Tissue collection

Renal samples were collected by perfusing rats with 4° C physiological saline through the left ventricle until the fluid ejected through the right atrium was clear. Animals were then perfused with 4% paraformaldehyde (PFA), and kidneys were collected. The right kidney was then weighed and stored for 48 h in 4% PFA.

### Preparation of tissue sections

Following fixation, right kidney samples were transferred to a 70% alcohol solution for 48 h at 4° C, after which they were dehydrated for 1 h using an ethanol gradient (70%, 80%, 90%, 100% I, 100% II). Samples were then treated for 15 minutes with a toluene alcohol solution (xylene/alcohol [v/v] = 1:1), followed by a 15 minute treatment with xylene and a 5 h paraffin-embedding step at 65° C (soft wax 2 h; hard wax 3 h). Serial coronal sections (5 μm thick) were then prepared from these paraffin-embedded tissues and were dried at 65° C.

### Hematoxylin and eosin (H&E) staining

Prepared tissue sections were de-paraffinized using xylene, rehydrated using an ethanol gradient (100%, 95%, 90%, 80%, 70%, 50%; 3 minutes each), and stained with hematoxylin for 5 minutes. Samples were then washed twice in water, treated with phosphate-buffered saline (PBS) (Sigma-Aldrich Co., St. Louis, USA) for 30 seconds, washed twice with water (30 seconds per wash), and dehydrated with an ethanol gradient (50%, 70%, 80%, 90%, 95%; 1 minute each). Samples were then stained with eosin for 30 seconds prior to further treatment with an ethanol gradient (95%, 100% I, 100% II; 1 minute each). Samples were then treated twice with xylene for 2 minutes, and were sealed with a neutral resin, dried, and visualized via light microscopy.

### Assessment of renal tissue ultrastructure

For ultrastructural analyses, animals were euthanized and perfused with 4% PFA as above. Kidneys were then collected and were fixed for 4 h using 4% glutaraldehyde. These tissues were then washed thrice using PBS (30 minutes per wash), followed by a 3-hour treatment with 1% citric acid. Next, samples were washed with PBS, dehydrates using an ethanol gradient (50%, 70%, 80%, 90%, 95%; 10 minutes). Next, propylene oxide was added for 20 minutes. Propylene oxide: Epon812 epoxy resin embedding agent (1: 1) soaked for 1h. Epon812 epoxy resin embedding agent soaked for 3h, 35° C, 45° C, 55° C polymerization for 12h. Next, semi-thin (1 μm) tissue sections were prepared, followed by the preparation of ultrathin (70 nm) sections that were stained using 0.4% uranyl acetate and 2 % citrate (10 minutes each). Samples were then visualized via TEM.

### Immunohistochemistry

Immunohistochemistry staining for CD31, CD34, IL-6, Fn, Collagen I, Talin-1, Paxillin, and α3β1 was performed using kidney sections, which were observed under a light microscope. Image-Pro plus was used to quantitatively evaluate CD31, CD34, IL-6, Fn, Collagen I, Talin-1, Paxillin, and α3β1 expression.

Immunohistochemical staining was all performed on 5-μm tissue sections. Briefly, the tissues were dewaxed and rehydrated. The tissue sections were incubated with 0.5% trypsin at 37° C for 20 minutes for antigen retrieval, and treated with a 3% methanol solution of hydrogen peroxide for 30 minutes to block endogenous peroxidase activity. Next, the sections were incubated with 3% bovine serum albumin in PBS for 30 minutes at room temperature to block non-specific protein binding, and then incubated with primary antibodies overnight, including (anti-CD31 at 1:200, anti-CD34 at 1:3000, anti-Collagen I at 1:6000, anti-Fibronectin at 1:1000, anti-Talin 1 at 1:6000, anti-Paxillin at 1:1000, anti-IL6 at 1:6000, anti-Integrin alpha 3 at 1:4000, and anti-Integrin beta1 at 1:8000). After incubating with biotinylated secondary antibody for 35 minutes, the tissue sections were incubated with Vectastain® Elite® ABC reagent (Vector Laboratories) for 35 minutes. Then 0.06% 3,3′-diaminobenzidine (Sigma, USA) plus hydrogen peroxide was used to detect peroxidase activity, and Carazzi's hematoxylin was used to stain the nucleus. For negative controls, primary antibody incubations were replaced with an incubation in non-immune serum. Image-Pro plus was used for quantitative analyses.

### Western blotting

The protein expression of podocin, nephrin and B7-1 in the kidney was detected by Western blotting. And the protein expression was quantified using Image Lab 3.0 (Beta3).

The kidney tissue was homogenized in a mixed buffer solution containing 100 mM NaCl, 20 mM Tris-HCl (pH 8.0), 1 mM EDTA, 10% NP-40 (v/v) and a protease inhibitor mixture (1: 100, Sigma). The BCA protein assay kit was used to measure the total concentration of the protein obtained above. The protein was then heated at 97° C for 5 minutes, separated by 10% SDS-PAGE and transferred onto a PVDF membrane (Millipore, USA). The membrane was incubated with specific primary antibodies (Anti-nephrin at 1:500, anti-podocin at 1:2000, and anti-B7-1 at 1:1000) and corresponding secondary antibodies. Protein bands were observed using an enhanced chemiluminescence reagent kit (Millipore, USA).

### Statistical analysis

SPSS v22.0 (SPSS Inc., IL, USA) was used for statistical testing. Data are means ± SD and were compared via one-way ANOVAs with post hoc LSD tests. P<0.05 was the significance threshold.
